# The Role of the Mechanotransduction Ion Channel Candidate Nanchung-Inactive in Auditory Transduction in an Insect Ear

**DOI:** 10.1523/JNEUROSCI.2310-17.2018

**Published:** 2018-04-11

**Authors:** Ben Warren, Tom Matheson

**Affiliations:** University of Leicester, Department of Neuroscience, Psychology and Behaviour, University Road, Leicester LE1 7RH, United Kingdom

**Keywords:** auditory transduction, chordotonal organ, desert locust, hearing, mechanotransduction ion channel, receptor current

## Abstract

Insect auditory receivers provide an excellent comparative resource to understand general principles of auditory transduction, but analysis of the electrophysiological properties of the auditory neurons has been hampered by their tiny size and inaccessibility. Here we pioneer patch-clamp recordings from the auditory neurons of Müller's organ of the desert locust *Schistocerca gregaria* to characterize dendritic spikes, axonal spikes, and the transduction current. We demonstrate that dendritic spikes, elicited by sound stimuli, trigger axonal spikes, and that both types are sodium and voltage dependent and blocked by TTX. Spontaneous discrete depolarizations summate upon acoustic stimulation to produce a graded transduction potential that in turn elicits the dendritic spikes. The transduction current of Group III neurons of Müller's organ, which are broadly tuned to 3 kHz, is blocked by three ion channel blockers (FM1-43, streptomycin, and 2-APB) that are known to block mechanotransduction channels. We investigated the contribution of the candidate mechanotransduction ion channel Nanchung-Inactive—which is expressed in Müller's organ—to the transduction current. A specific agonist of Nanchung-Inactive, pymetrozine, eliminates the sound-evoked transduction current while inducing a tonic depolarizing current of comparable amplitude. The Nanchung-Inactive ion channels, therefore, have the required conductance to carry the entire transduction current, and sound stimulation appears not to open any additional channels. The application of three mechanotransduction ion channel blockers prevented the pymetrozine-induced depolarizing current. This implies that either Nanchung-Inactive is, or forms part of, the mechanotransduction ion channel or it amplifies a relatively small current (<30 pA) produced by another mechanotransduction ion channel such as NompC.

**SIGNIFICANCE STATEMENT** The mechanically activated ion channel underpinning hearing is not known. We have pioneered intracellular patch-clamp recordings from locust auditory neurons to unravel the role of the candidate mechanotransduction ion channel Nanchung-Inactive in auditory transduction in insects.

## Introduction

The startling variety of auditory receptors found in insects provides a rich resource for the comparative analysis of auditory mechanotransduction. For instance, insect “ears” have been used to understand traveling waves that are necessary for frequency discrimination (Nowotny et al., 2010), otoacoustic emissions (Kössl and Boyan, 1998; Möckel et al., 2007) that can be used to assess hearing impairment (Kemp, 2002), and the roles of specific ion channels in auditory transduction (Göpfert et al., 2006). Despite these advances, the investigation of insect auditory transduction has been hampered by the tiny size and inaccessibility of the auditory neurons. Recordings of the mechanical-to-electrical transduction events that lead to spike generation in the auditory neurons themselves are sparse (Hill, 1983a,b; Oldfield and Hill, 1986). These early studies revealed that quiescent insect auditory neurons exhibit discrete depolarizations that are thought to represent the first mechanical-to-electrical event in the dendritic cilium (Hill, 1983a). Upon acoustic stimulation, these discrete depolarizations summate to produce a graded transduction potential, which, if large enough, generates an apical spike that propagates toward the soma to elicit a larger basal spike (Hill, 1983b; Oldfield and Hill, 1986).

More recent attention has focused on the cilium, where transduction is thought to take place and where two transient receptor potential (TRP) mechanotransduction ion channel candidates, NompC and the heteromer Nanchung-Inactive, have been localized to the tip and the proximal cilium, respectively (Gong et al., 2004; Lee et al., 2010). In *Drosophila*, the direct coupling of auditory neurons to the antennae (which act as sound receivers) has allowed measurement of the coordinated opening and closing of thousands of mechanotransduction ion channels in the cilia. These channel state changes are detected as nonlinear mechanical displacements of the antennae during sound stimulation (Albert et al., 2007). In *nompC* mutants, this nonlinear mechanical amplification is abolished, but in *nanchung* or *inactive* mutants it is amplified. This led to a “NompC model,” which postulates NompC, localized to the tips of auditory neuron cilia, as the mechanotransduction ion channel. In this model, Nanchung-Inactive propagates a NompC-mediated depolarization along the cilium, toward the dendrite (Göpfert et al., 2006). In insects, NompC is a leading contender for the transduction channel. There is strong evidence that the NompC subfamily channels, such as TRP-4, form mechanotransduction ion channels in *Caenorhabditis elegans* (Kang et al., 2010). Calcium signals are abolished in *nompC*-null mutations in *Drosophila* sound-sensitive neurons (Effertz et al., 2011) and in larval multidendritic neurons (Cheng et al., 2010), and finally, NompC is mechanically sensitive when expressed ectopically or in heterologous cells (Gong et al., 2013; Yan et al., 2013).

In a complementary approach, indirect measurements of the transduction current were made in *Drosophila* using intracellular recordings of the postsynaptic giant fiber neurons onto which hundreds of antennal mechanosensory neurons are electrically synapsed (Lehnert et al., 2013). The sound-induced synaptic currents in the giant neurons were reduced in *nompC* loss-of-function mutants but were abolished in *nanchung* mutants. This implicated Nanchung-Inactive as the mechanotransduction ion channel, whereas NompC was postulated to regulate the tension delivered to the cilium. The electrotonically distant location of the recording site in the postsynaptic neurons (rather than the auditory neurons themselves) leaves open the possibility that a NompC current in the distal cilium triggers Nanchung-Inactive currents as proposed by the NompC model. In contrast to NompC, Nanchung-Inactive channels are mechanically insensitive when expressed in heterologous cells. Although heterologous expression of Nanchung or Inactive was claimed (although not shown) to confer hypotonically activated currents (Kim et al., 2003; Gong et al., 2004), this could be due to intrinsic properties of the cells themselves (Nesterov et al., 2015).

In this study, we perform whole-cell patch-clamp recordings from the auditory neurons of Müller's organ of the desert locust. We use electrophysiological and pharmacological tools to quantitatively investigate dendritic and axonal spike types and the transduction current, and analyze the role of Nanchung-Inactive in mechanotransduction in auditory neurons of the desert locust.

## Materials and Methods

### 

#### 

##### Locust husbandry.

Desert locusts (*Schistocerca gregaria*) from a long-term culture at the University of Leicester were reared in crowded conditions (phase gregaria) on a 12 h light/dark cycle at 36°C and 25°C, respectively. Locusts were fed on a combination of fresh wheat and bran. Male locusts between 10 and 20 d after imaginal molt were used for all experiments.

##### Dissection of Müller's organ and isolation of Group III auditory neurons.

Whole ears, including Müller's organ attached to the internal side of the tympanum, were dissected from the first abdominal segment by cutting around the small rim of cuticle surrounding the tympanum with a fine razor blade. Trachea and the auditory nerve (Nerve 6) were cut with fine scissors (5200-00, Fine Science Tools), and the trachea and connective tissue were removed with fine forceps. The ear was secured, inner side up, into a 2-mm-diameter hole in a Perspex divider using an insect pin pushed through the anterior rim of cuticle and into 2 mm of Sylgard (184 Silicone Elastomer, Dow Corning) on the base of a 30-mm-diameter Petri dish. The tympanum in its holder was positioned at an angle of 30° off vertical to observe Group III neurons of Müller's organ from above, illuminated by light transmitted through the tympanum. A watertight seal was made between the ear cuticle and the divider hole with dental glue (Protemp 4, 3M ESPE), and Nerve 6 was secured into the glue at the ventral border of the tympanum. This preparation allowed the perfusion of saline to the internal side of the tympanum, which is necessary for water-immersion optics for visualizing Müller's organ and permitting the auditory neurons to be patch clamped, and concurrent acoustic stimulation to the dry external side of the tympanum. The inside of the tympanum, including Müller's organ, was constantly perfused with extracellular saline.

To expose Group III auditory neurons for patch-clamp recordings, a solution of collagenase (0.5 mg/ml) and hyaluronidase (0.5 mg/ml; C5138, H2126, Sigma-Aldrich) in extracellular saline was applied onto the medial–dorsal border of Müller's organ through a wide (12 μm) patch pipette to digest the capsule enclosing Müller's organ and the Schwann cells surrounding auditory neuron somata. Gentle suction was used through the same pipette to remove the softened material and expose the somatic membrane of Group III auditory neurons. The somata were visualized with an upright microscope (BH-2, Olympus) using a water-immersion objective (40×, 1.0 numerical aperture, 2.5 mm working distance; W Plan-APOCHROMAT, Zeiss) and differential interference contrast optics.

##### Electrophysiological recordings and isolation of the transduction current.

Electrodes with tip resistances between 3 and 4 MΩ were fashioned from borosilicate class (inner diameter, 0.86 mm; GB150–8P, Science Products) with a vertical pipette puller (PC-10, Narishige). Recording pipettes were filled with intracellular saline containing the following (in mm): 190 K-aspartate, 4 NaCl, 2 MgCl_2_, 1 CaCl_2_, 10 HEPES, and 10 EGTA.

During experiments, Müller's organs were continuously perfused with extracellular saline containing the following (in mm[scap]): 185 NaCl, 10 KCl, 2 MgCl_2_, 2 CaCl_2_, 10 HEPES, 10 trehalose, and 10 glucose. To reduce external sodium to assess the effect on dendritic and axonal spikes NaCl was reduced to 7 mm and replaced with 178 choline chloride. To block spikes necessary for analyzing the summation of discrete depolarizations into the transduction current 90 mm tetrodotoxin (TTX) citrate was added to the extracellular saline. To isolate the transduction current for quantitative analysis, in addition to TTX, 20 mm tetraethylammonium (TEA) was added to the pipette saline to block potassium channels. In this case, K-aspartate was reduced to 170 mm to maintain the same osmolality.

Together, TTX and TEA increased the transduction current from a median of 106–144 pA (*n* = 20, *N* = 18; *n* = 19, *N* = 17; for normal saline and TTX plus TEA saline, respectively; Student's unpaired two-tailed *t* test, *p* = 0.035). The saline was adjusted to pH 7.2 using NaOH. The osmolality of the intracellular and extracellular salines were 417 and 432 mOsm, respectively.

Mechanotransduction ion channel blockers were used at the following concentrations: gadolinium (III) chloride hydrate, 300 μm (catalog #450855, Sigma-Aldrich); amiloride hydrochloride hydrate, 30 μm (catalog #A7410, Sigma-Aldrich); Ruthenium red, 30 μm (catalog #ABE7684, Source Bioscience UK Limited); SKF-96365, 100 μm (catalog #S7809, Sigma-Aldrich); FM1-43 (also known as Synaptogreen C4), 3 μm (catalog #S6814, Sigma-Aldrich); dihydrostreptomycin sesquisulfate, 100 μm (see Fig. 6) or 300 μm (see Fig. 8; catalog #D7253, Sigma-Aldrich); and 2-aminoethoxydiphenyl borate (2-APB), 100 μm (catalog #1798, Cambridge Bioscience). The mechanotransduction ion channel blockers were perfused at least 15 min before recordings. To open Nanchung-Inactive ion channels, fast-acting pymetrozine PESTANAL, 30 μm (catalog #46119, Sigma-Aldrich) was dissolved in extracellular saline and perfused over the preparation for 2 min.

Whole-cell voltage-clamp and current-clamp recordings were performed with an EPC10-USB patch-clamp amplifier (HEKA Elektronik) controlled by the program Patchmaster (version 2x90.2, HEKA Elektronik) running under Microsoft Windows. Electrophysiological data were sampled at 50 kHz. Voltage-clamp recordings were low-pass filtered at 2.9 kHz with a four-pole Bessel filter. Compensation of the offset potential and capacitive current were performed using the “automatic mode” of the EPC10 amplifier. The calculated liquid junction potential between the intracellular and extracellular solutions was also compensated during recordings (normal saline and saline with TTX, 13.5 mV; when the pipette saline contained TEA, 15.6 mV; calculated with Patcher's-PowerTools plug-in from www3.mpibpc.mpg.de/groups/neher/index.php?page=software). The liquid junction potential of the low extracellular sodium solution was not compensated for when washing from and to normal saline (see Fig. 4). Series resistance was compensated between 33% and 70% with a time constant of 100 μs. For current-clamp recordings, series resistance was compensated by bridge balance set at 44%. Data were analyzed in Igor Pro (version 6.3.7.2; RRID:SCR_000325) with Patcher's Power Tools (RRID:SCR_001950).

##### Sound pressure level calibration and sound stimulation.

We measured the voltage output of an amplified microphone (Clio PRE-01 and Clio MIC-03, Audiomatica) signal in response to a 94 dB SPL 1 kHz tone from a sound level calibrator (catalog #Cal73, Brüel & Kjær). This allowed us to calculate the decibel SPL based on the voltage output of the microphone. We then placed the microphone where the excised locust ear was placed during experiments. We adjusted the voltage output delivered to a speaker (Visaton FR 10 HM, RS Components), which was amplified through a custom audio amplifier suspended 8 cm from the preparation to play pure tones with the desired decibel SPL.

##### Staining and confocal microscopy.

To stain Group III auditory neurons, recording electrodes were filled with Neurobiotin (1% m/v; catalog #SP-1120, Vector Laboratories) dissolved in intracellular saline. To aid the diffusion of Neurobiotin into the neurons, a positive current of ∼200 pA was injected for ∼30 min after recordings. Directly after staining, Müller's organs were fixed overnight at 5°C in 4% paraformaldehyde (catalog #P6148, Sigma-Aldrich) dissolved in PBS. Müller's organs were washed three times in PBS then gently shaken at room temperature for 20 min in a mixture of collagenase (0.5 mg/ml) and hyaluronidase (0.5 mg/ml). They were washed three times in PBS (3× 10 min) then gently shaken at room temperature in 0.2% m/v Triton X-100 dissolved in PBS (2× 60 min). Müller's organs were then gently shaken in 20 μg/ml Dylight 488 streptavidin (catalog #SA-5488, Vector Laboratories) and 0.05 mg/ml DAPI (catalog #D9542, Sigma-Aldrich) in PBS overnight at 5°C, washed three times in PBS (3× 10 min), dehydrated in an ethanol series and cleared in methyl salicylate (catalog #M6752, Sigma-Aldrich).

Fluorescence images (pixel size, 0.31 × 0.31 μm; *z* stacks at 0.31 μm resolution) were captured with a confocal microscope (model FV1000 CLSM, Olympus) equipped with Plan-UPlanSApo 10× (0.4 numerical aperture) and 20× (0.75 numerical aperture) lenses. Fluorescence emission of Dylight 488 was stimulated with a 488 nm argon laser and collected through a 505–530 nm bandpass filter, and fluorescence emission of DAPI was stimulated with a 405 nm UV laser diode and collected through a 490 low-pass filter. Confocal images were adjusted for contrast and brightness, overlaid, and stacked in ImageJ (version 1.51, National Institutes of Health; RRID:SCR_003070). The ImageJ plugin Simpler Neurite Tracer (RRID:SCR_002074) was used to determine the distance from the center of the soma to the dendrite dilation (see Fig. 7*A*).

##### Extraction and sequencing of *nompC*, *inactive*, and *nanchung* transcripts.

**Figure 1.**
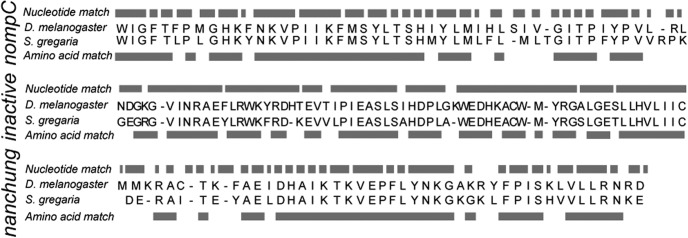
Sequence comparison between *S. gregaria* Müller's organ-specific homologs of *D. melanogaster nompC*, *inactive*, and *nanchung*. The nucleotide sequence similarity was 69%, 78%, and 68%, respectively, for transcripts of 147, 207, and 141 nucleotides bases in length. Amino acid sequence similarity was 66%, 75%, and 70%, respectively.

Transcripts of *S. gregaria nompC*, *inactive*, and *nanchung* homologs were determined from whole-brain and thoracic ganglia transcriptome analysis and comparison with *Drosophila melanogaster* genomic sequences (Fig. 1). We used *S. gregaria* transcriptome sequences to design primers to amplify locust *nompC*, *inactive*, and *nanchung* transcripts and establish their expression in Müller's organ. For these experiments, *S. gregaria*, 1–3 d after imaginal molt, was purchased from Blades Biological. One hundred twenty Müller's organs were extracted from 60 locusts by grasping the Müller's organ through the tympanum with fine forceps and pulling it out. Müller's organs were placed in an Eppendorf tube on dry ice, RNA was extracted (concentrations between 20 and 50 ng/μl; RNeasy Plus Micro Kit, Qiagen) and cDNA was synthesized (Tetro cDNA Synthesis Kit, Qiagen). *nompC*, *inactive*, and *nanchung* transcripts were amplified using PCR (My TaqDNA Polymerase Kit, Qiagen) and primers designed from in-house transcriptome sequences and synthesized by Sigma-Aldrich. Gel electrophoresis of the PCR products was used to confirm that their length matched that of the target transcripts. PCR products were purified (QIAquick PCR Purification Kit, Qiagen) and then sequenced by GATC Biotech using the Sanger sequencing method. The sequenced Müller's organ-specific *nompC*, *inactive*, and *nanchung* transcripts had 66%, 75%, and 70% sequence similarity to amino acid sequences of *D. melanogaster* (Fig. 1). This establishes the expression of locust homologs of *nompC*, *inactive*, and *nanchung* in Müller's organ. The analysis does not preclude the possibility that other channels are present, and does not demonstrate that *NompC*, *inactive*, and *nanchung* are expressed specifically in neurons.

##### Experimental design and statistical analysis.

Throughout the manuscript, *n* refers to the number of recorded neurons and *N* refers to the number of Müller's organ preparations used to achieve these recordings (i.e., *n* = 10, *N* = 6 means that 10 neurons were recorded from 6 Müller's organs). Spreads of the data indicated by “±” are all 1 SD. We made sufficient recordings to maintain a statistical power of >0.95. To provide the first quantitative analysis of spike types in insect auditory neurons, we used *n* numbers of 9–10 for analyzing the effect of low sodium on spike amplitude and 5–6 for analyzing the effect of TTX on spike amplitude. The effect of low sodium and TTX was estimated to decrease spike currents to at least a half and a third (respectively) of their original amplitude, so the effect sizes were 2 and 3, respectively, when we performed our power analysis. To determine the tuning of the Group III neurons and their responsiveness to the sound intensity, we used sample sizes of 7 and 11, respectively. The effect of the mechanotransduction ion channel blockers took at least 10 min, and the sound-evoked transduction current had significant rundown and was abolished ∼10 min after establishing a whole-cell recording. We therefore assessed the ability of mechanotransduction ion channel blockers (see Fig. 7*C*) using unpaired recordings, and for the corresponding statistical analyses we required higher sample sizes of 10–20 to maintain a statistical power of >0.95.

To control for multiple comparisons of paired recordings (see Figs. 4, 5), we used Sidak's correction. We did not assume sphericity of the data as the variance of the spike amplitude would tend to zero when the spike amplitude tended to zero either by low extracellular sodium (see Fig. 4) or through TTX block (see Fig. 5). We therefore used the Geisser–Greenhouse correction. To test for correlation between the dendrite length and the transduction current amplitude (see Fig. 7*B*), we fitted a linear regression to the data, which resulted in the following parameters [*R*^2^ = 0.00442, *p* = 0.7806 (likelihood of a correlation significantly different from 0) and equation of the line *y* = 0.1683 *x* + 136.96]. Analysis of the relationship between transduction current amplitude and mechanotransduction ion channel blockers was made through one-way unpaired ANOVA with Bonferroni's multiple-comparison test where each blocker was compared against control recordings (see Fig. 7*C*). To correct for multiple comparisons, we used Sidak's correction. The effect of the fast-acting Nanchung-Inactive channel agonist pymetrozine was analyzed using a two-tailed paired *t* test (see Figs. 8, 9). All statistical analyses were performed using GraphPad Prism software (version 7; RRID:SCR_000306).

## Results

### Auditory neurons have two distinct spike types and a graded transduction potential.

**Figure 2.**
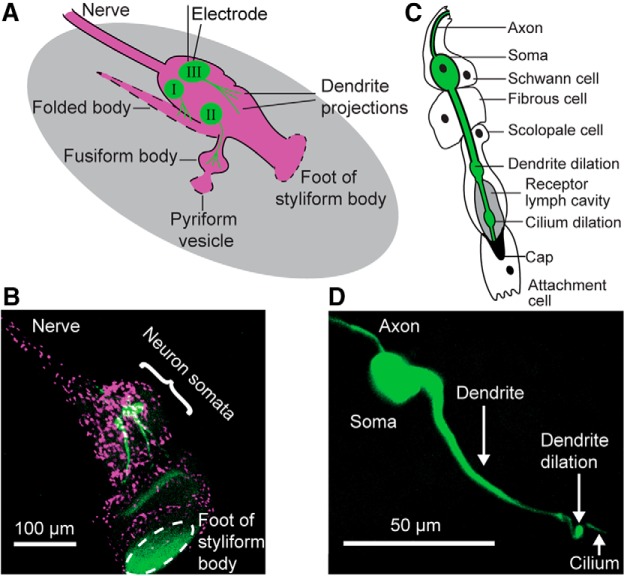
Morphology of Müller's organ and the auditory neurons. ***A***, Schematic of Müller's organ (magenta) viewed from the inner aspect, and the three populations of sensory neurons (green, I–III) with their dendritic projections. The gray oval represents the tympanum. Dashed parts of the outline indicate points of attachment of Müllers organ to the tympanum. ***B***, Double staining of the nuclei of cells of Müller's organ (magenta, DAPI) and three Group III auditory neurons individually stained with neurobiotin/Dylight 488 strepavidin (green). There is some nonspecific green fluorescence at the point of attachment of the organ to the tympanum. ***C***, Schematic of one scolopidium, consisting of an auditory neuron (green) enclosed in a Schwann cell, a fibrous cell, and scolopale cell, which enclose the soma and axon, the basal dendrite, and the distal dendrite and cilium, respectively. The cilium of the sensory neuron is embedded in an electron-dense cap produced by a distal attachment cell. The neuronal dendrite has a prominent dilation close to the base of the cilium. From the distal dendrite extends an apical cilium, which also possesses a dilation that divides it into proximal and distal regions. The receptor lymph cavity enclosed by the scolopale cell that bathes the cilium is shaded. ***D***, Neurobiotin-streptavidin staining of a Group III auditory neuron *in situ* reveals the dendrite dilation and apical cilium.

Intracellular patch-clamp recordings were used to understand dendritic and axonal spike types (previously classified as apical and basal spikes; Hill, 1983b; Oldfield and Hill, 1986) in auditory neurons of Müller's organ of the locust. We performed whole-cell patch-clamp recordings solely from the somata of Group III neurons (Fig. 2*A*,*B*), which represent ∼46 of the 80 neurons of Müller's organ (Jacobs et al., 1999). Group III neurons are attached to the elevated process and styliform body and are most sensitive to frequencies of 3–4 kHz. In addition to these, 20 Group I neurons are attached to the folded body and are most sensitive to frequencies of ∼0.5 and ∼1.5 kHz. A further 12–14 high-frequency, sensitive (12–25 kHz) Group II neurons are attached to the fusiform body (Jacobs et al., 1999). Together, the auditory neurons of Müller's organ are attached to distinct parts of the tympanum to exploit the spatial patterns of the tympanum of frequency-specific displacements to detect and encode frequencies between 0.2 and 40 kHz. Single auditory neurons are surrounded by scolopale, attachment, fibrous, and Schwann cells (Fig. 2*C*; Gray, 1960). The neurons have a distal dendrite dilation close to the base of the terminal mechanosensory cilium (Fig. 2*D*).

**Figure 3.**
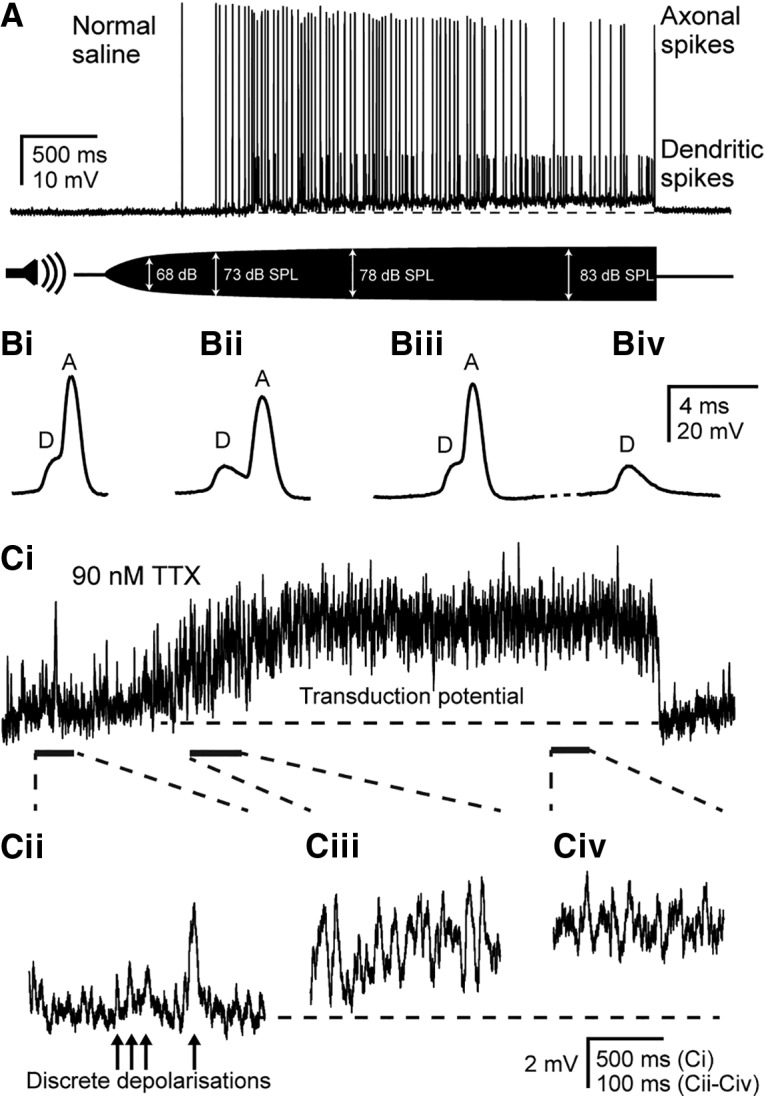
Dendritic spikes trigger axonal spikes, and discrete depolarizations summate to produce a graded transduction potential. ***A***, Current-clamp recording from the soma of a Group III neuron at a resting membrane potential of − 75 mV. The cell is depolarized and produces both small-amplitude dendritic spikes and large-amplitude axonal spikes in response to a 3 kHz tone that is amplitude ramped from 0 to 83 dB SPL. ***Bi–Biv***, Expanded view of spikes in ***A*** at progressive time points during the recording. Dendritic spikes precede and give rise to axonal spikes (***Bi–Biii***), but axonal spikes can fail, leaving only the underlying dendritic spike (***Biv***). ***Ci***, Transduction potential in response to the same amplitude-ramped 3 kHz tone as shown in ***A***, but with 90 nm TTX to block spikes, measured at resting potential (note different vertical scale compared with ***A***). ***Cii–Civ***, Expanded views of transduction potential at the time points indicated by dashed lines and solid bars, showing discrete depolarizations and their summation to produce a graded transduction potential. Horizontal dashed lines in ***A*** and ***Ci*** indicate the resting membrane potential of −75 and −69 mV, respectively.

The median resting membrane potential of Group III neurons was −70 mV (mean ± SD, −64.5 ± 18.4 mV; *n* = 25, *N* = 21), median capacitance was 22.5 pF (mean ± SD, 23.7 ± 6.3 pF; *n* = 23, *N* = 19), and median membrane resistance was 43.6 MΩ (mean ± SD, 64.9 ± 56.7 MΩ; *n* = 23, *N* = 19). An intensity-ramped 3 kHz pure tone was used to acoustically stimulate the tympanum onto which Müller's organ is attached (Fig. 3*A*). As the sound intensity increased, this resulted in a small (3 mV) prolonged depolarization and trains of spikes of two distinct amplitudes in whole-cell patch-clamp recordings of Group III auditory neurons (Fig. 3*A*). In this example, the amplitude threshold for triggering spikes was 73 dB SPL. Note that in these *ex vivo* conditions the tympanum is backed by saline, and not air, as is the case *in vivo*, thus requiring higher SPLs to elicit comparable responses. The larger spikes had a baseline to peak amplitude of up to 75 mV (median, 45 mV; *n* = 23, *N* = 18) and peaked at −6 mV (median, −23 mV; *n* = 22, *N* = 17). These large spikes are termed “axonal spikes” as they are thought to originate in the axon initial segment. In contrast, the smaller dendritic spikes are thought to originate at the apex of the dendrite.

At normal resting potential, the majority of neurons (9 of 15; *n* = 15, *N* = 13) had a clear inflection on the r**i**sing phase of their axonal spikes, and all neurons displayed an inflection under nonresting holding potentials (15 of 15; *n* = 15, *N* = 13; inflection was determined by a decrease in the rate of depolarization of at least 0.5 μA/s). Under prolonged (>0.5 s) stimulation with a 3 kHz tone, axonal spikes eventually failed. However, the small-amplitude dendritic spikes persisted and were of an amplitude similar to that of the inflection on the axonal spikes (Fig. 3*Bi–Biv*). Thus, the smaller-amplitude inflections that precede axonal spikes resulted from smaller underlying dendritic spikes with a maximum baseline-to-peak amplitude of 21 mV (median, 11 mV; *n* = 9, *N* = 8) and peak up to −52 mV. The dendritic and axonal spikes summate to determine the overall spike amplitude (Fig. 3, compare *Bi*, *Bii*).

To analyze the graded 3 mV depolarization elicited by the tone (Fig. 3*A*) thought to be the receptor potential, dendritic and axonal spikes were blocked using 90 nm TTX (Fig. 3*Ci*). This revealed that the graded depolarization is made up of summated discrete depolarizations that were also present in the absence of acoustic stimulation (Fig. 3*Cii*). Upon stimulation with an amplitude-ramped 3 kHz tone, the frequency of these discrete depolarizing bumps increased and summated to produce a graded transduction potential (Fig. 3*Ciii*,*Civ*). The transduction potential increased in amplitude >68 dB SPL before spikes were elicited at 73 dB SPL. In summary, discrete depolarizations due to the sound-activated transduction potential summate to trigger dendritic spikes, which, in turn, elicit axonal spikes.

### Dendritic and axonal spikes are sodium and voltage dependent and blocked by TTX

**Figure 4.**
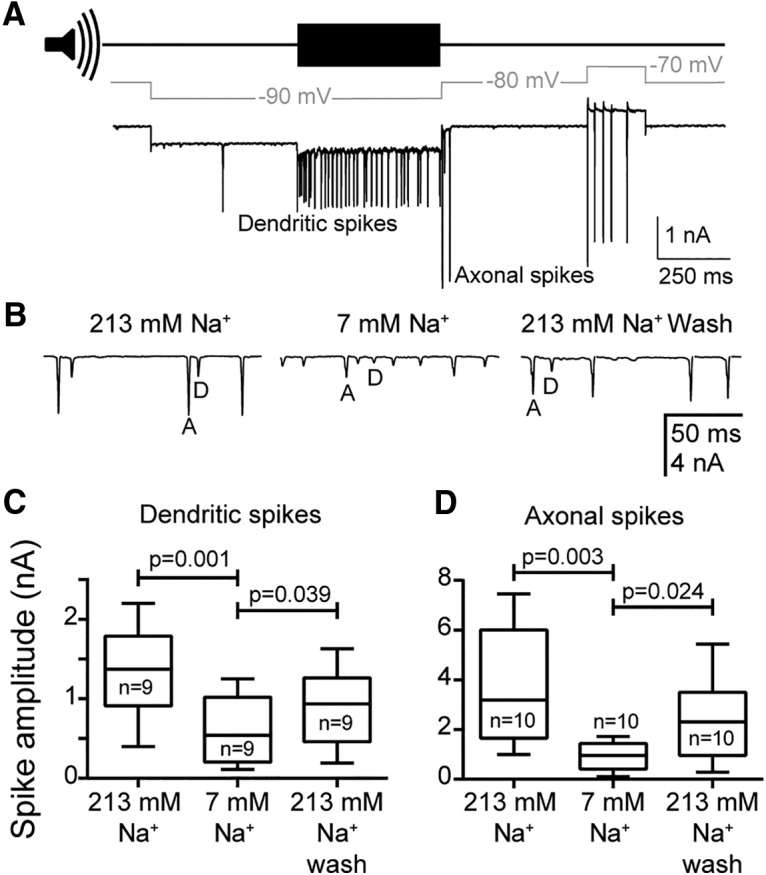
The amplitude of dendritic and axonal spikes depends on extracellular sodium. ***A***, Voltage-clamp protocol to obtain dendritic and axonal spikes. The resting membrane potential of the cell was −67 mV, and the baseline holding potential was −80 mV. Note that on release from hyperpolarizing steps, the cell sometimes fired axonal spikes. ***B***, Example from one neuron showing the reversible effect of low (7 mm) extracellular sodium on the spike amplitude of dendritic (D) and axonal (A) spikes. ***C***, ***D***, Quantification of the effect of extracellular sodium concentration on the amplitude of dendritic spikes (***C***; *n* = 9, *N* = 9) and axonal spikes (*D*; *n* = 10, *N* = 10). Median, interquartile ranges and ranges of the data are plotted.

To elicit dendritic spikes alone, the neurons were first hyperpolarized to −10 to −20 mV relative to the resting potential (i.e., to approximately −90 mV) to prevent the spike threshold for axonal spikes being reached, and then acoustically stimulated (Fig. 4*A*; Oldfield and Hill, 1986). Auditory stimulation generated a burst of dendritic spikes in the absence of larger axonal spikes (Fig. 4*A*). To elicit axonal spikes alone, neurons were subsequently voltage clamped 10 mV above the resting potential (i.e., at approximately −60 to −70 mV) to depolarize the neuron above the axonal spike threshold (Fig. 4*A*). In this case, dendritic spikes were not elicited due to the electrotonically distant dendritic spike initiation site where the amplitude of the voltage step was attenuated by cable properties of the dendrite. This generated a burst of axonal spikes in the absence of smaller underlying dendritic spikes (Fig. 4*A*). We used voltage clamp instead of current clamp because we were able to more reliably exploit the electrotonically close and distant spike initiation sites for axonal and dendritic spikes to elicit spike only of one type. Dendritic and axonal action currents were measured first with Müller's organ bathed in 213 mm extracellular sodium, then during perfusion with 7 mm extracellular sodium (sodium chloride replaced with choline chloride), and finally during the wash back to 213 mm extracellular sodium (Fig. 4*B*). Under baseline 213 mm conditions, dendritic spikes had a median amplitude of 1.4 nA (Fig. 4*C*), whereas axonal spikes had an amplitude of 3.2 nA (Fig. 4*D*). Under 7 mm conditions, dendritic spikes had a median amplitude of 0.56 nA (Fig. 4*C*) and axonal spikes had a median amplitude of 0.96 nA (Fig. 4*D*). Both spike type amplitudes partially recovered during a subsequent wash (median dendritic amplitude, 0.94 nA; axonal amplitude, 2.3 nA; Fig. 4*B–D*), which represents a recovery of median spike amplitudes to 68% and 72% of the control values for dendritic and axonal spikes, respectively. The amplitudes of dendritic and axonal spikes were therefore reversibly dependent on extracellular sodium (one-way ANOVA, Sidak's multiple-comparisons test, with Geisser–Greenhouse correction, between spikes before and during low sodium and during low sodium and wash; Fig. 4*C*,*D*).

**Figure 5.**
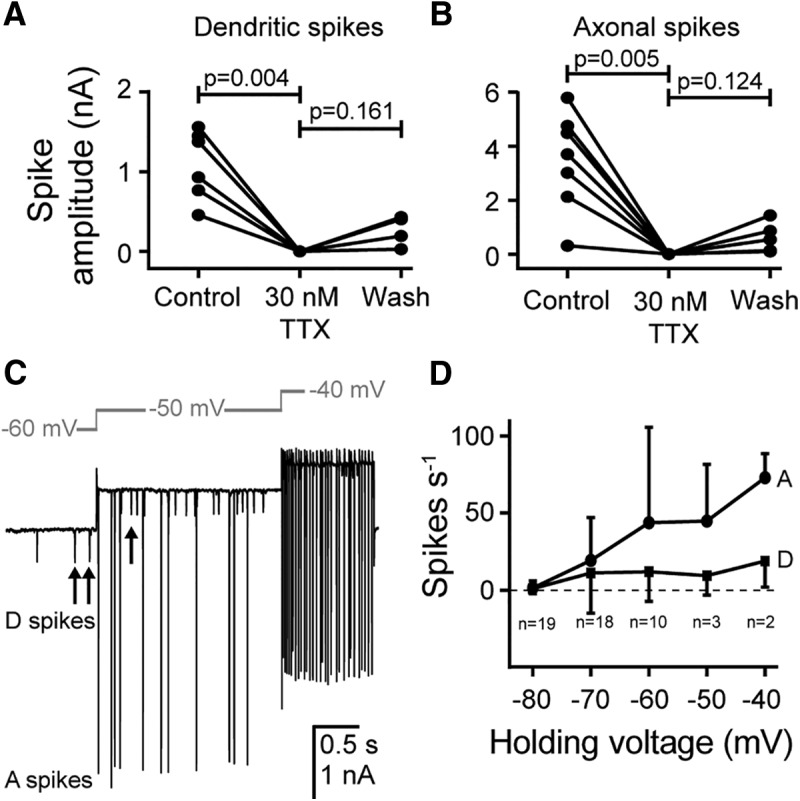
Dendritic and axonal spikes are blocked by TTX and are voltage dependent. ***A***, ***B***, Quantification of the effect of 30 mm TTX on the amplitude of dendritic spikes (***A***; *n* = 6, *N* = 6) and axonal spikes (***B***; *n* = 7, *N* = 7). TTX completely abolished both dendritic and axonal spikes with some recovery. ***C***, In the absence of acoustic stimulation, the frequencies of spontaneous dendritic and axonal spikes are increased as the cell is held at progressively less negative potentials. ***D***, Quantification showing that both dendritic (D) and axonal (A) spikes are triggered by an increase in holding voltage to less negative potentials (*n*, *N* = 19, 17; 18, 16; 10, 10; 3, 3; and 2, 2 for −80; −70; −60; −50; and −40 mV, respectively; the SD is shown in one direction only for figure clarity).

To quantitatively determine the role of voltage-gated sodium channels in generating both spike types, we applied 30 nm TTX. This completely blocked both dendritic (Fig. 5*A*) and axonal (Fig. 5*B*) spike currents from median amplitudes of and 1.15 nA (*N* = 6) and 3.69 nA (*N* = 7), respectively (one-way ANOVA, Sidak's multiple-comparisons test between spikes before and during TTX, and during TTX and wash, with Geisser–Greenhouse correction; Fig. 5*A*,*B*). The block was partially reversed in some preparations by subsequent wash in saline with recovery to median spike amplitudes for dendritic and axonal spikes of 0.11 and 0.13 nA, respectively, representing a 10% and 3% recovery (Fig. 5*A*,*B*). Although TTX is a reversible blocker for voltage-gated sodium channels (Narahashi et al., 1964), a complete recovery of spike amplitude was not achieved in these experiments, probably because the neurons are embedded within Müller's organ. To examine the voltage dependence of the dendritic and axonal spikes, neurons were voltage clamped at a range of holding potentials between −80 and −40 mV (Fig. 5*C*). Both the dendritic and the axonal spike rates increased for more positive holding potentials (Fig. 5*D*).

### Isolation of the transduction current

**Figure 6.**
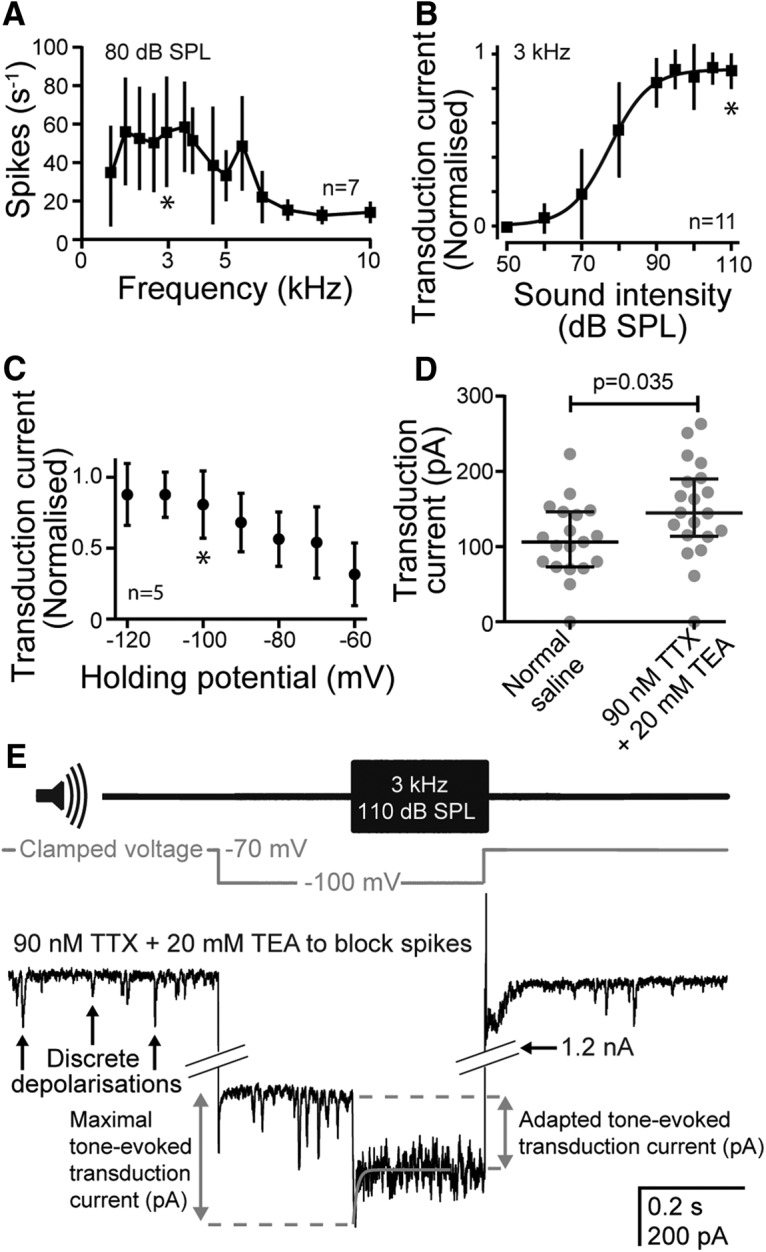
Characterization of Group III auditory neuron response to sound. ***A***, Frequency tuning of Group III auditory neurons to 80 dB SPL pure tones (*n* = 7, *N* = 4). The asterisk indicates the frequency (3 kHz) that was used in all subsequent characterizations of the transduction current. ***B***, Dependence of the transduction current on the sound intensity of a 3 kHz pure tone (*n* = 11, *N* = 11). The asterisk indicates the SPL (110 dB) that was used in all subsequent characterizations of the transduction current. ***C***, Dependence of the transduction current on the holding potential. The asterisk indicates the holding potential used in all subsequent characterization of the transduction current. ***D***, Dependence of the transduction current on the presence of 90 nm extracellular TTX and 20 mm intracellular TEA. ***E***, Voltage-clamp protocol used to maximize the transduction current using extracellular application of 90 nm TTX and intracellular application of 20 mm TEA to block sodium and potassium conductance; voltage clamp to −100 mV (gray trace); and a 3 kHz tone at 110 dB SPL (top trace). The transduction current was maximal at tone onset before rapidly adapting to an adapted transduction current (gray dashed lines). Adaptation of the transduction current was fitted with a first-order exponential equation (gray line overlying the transduction current. For ***A–C***, standard deviation was plotted and for ***D***, median and interquartile ranges are overlaid on the data points.

The auditory mechanotransduction current was isolated using a combination of optimized acoustic stimulation, pharmacological block of unwanted conductances (see Materials and Methods), and hyperpolarization to increase the electrochemical driving force. The Group III neurons were most sensitive to a median frequency of 3 kHz (mean ± SD, 3.2 ± 0.73 kHz; *n* = 7, *N* = 4) when they produced ∼60 spikes/s at 80 dB SPL (Fig. 6*A*). When stimulated at 3 kHz, the transduction current was minimal at up to 50 dB SPL, increased sigmoidally between 50 and 90 dB SPL, and saturated at >100 dB SPL (Fig. 6*B*). In all further characterizations of the transduction current, stimuli were delivered at 3 kHz, 110 dB SPL. The amplitude of the sound-elicited transduction current was increased by hyperpolarization of the neuron to −100 mV due to an increase in the electrochemical driving force at the site of transduction (Fig. 6*C*). The transduction current (with cells clamped at −100 mV) was further increased by block of sodium and potassium conductances using TTX and TEA from a median amplitude of 105–144 pA (Fig. 6*D*; see Materials and Methods). Thus, we maximized the transduction current through a combination of an optimized acoustic stimulus, hyperpolarization of the neuron, and pharmacological block of unwanted conductances. Figure 6*E* illustrates this protocol that was used to measure the maximal tone-evoked transduction current. TTX and TEA were first applied to block spikes, and the cell was clamped at −60 mV (Fig. 6*E*, gray trace) to record ongoing transduction currents, including discrete depolarizations (Fig. 6*E*, black arrows). The cell was then clamped at −100 mV for 250 ms to enhance the electrochemical driving force, before a 3 kHz tone (Fig. 6*E*, top trace) was applied for a further 250 ms to elicit the auditory transduction current (Fig. 6*E*, gray dashed lines). The auditory transduction current reached a transient maximum at tone onset (“maximal tone-evoked transduction current”; Fig. 6*E*, left, double-headed arrow) and then adapted back to a sustained level, the “adapted tone-evoked transduction current” (Fig. 6*E*, right, double-headed arrow). At the end of the tone, the neuron was returned to a −60 mV holding potential.

### Transduction current is not dependent on dendrite length and is blocked by three of seven known mechanotransduction ion channel blockers

**Figure 7.**
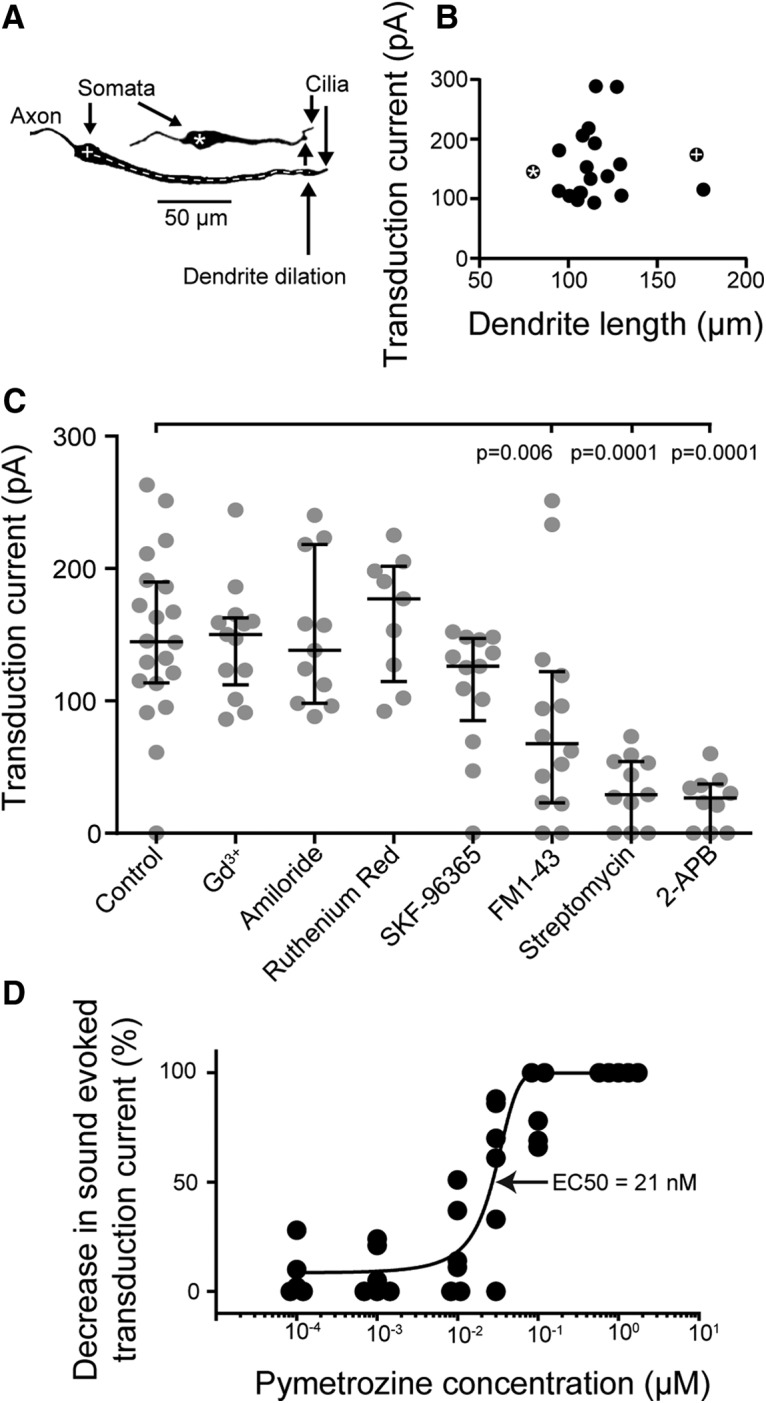
Characterization of the transduction current. ***A***, Neurobiotin-strepavidin staining of two auditory neurons. The white asterisk and plus symbols that label the neurons (in the somata) correspond to the data points in ***B***. Dendrite length was measured from the center of the soma to the dendrite dilation (white dashed line). ***B***, Dependence of the adapted transduction current on the length of the dendrite. ***C***, Effect on adapted transduction current amplitude of a range of mechanotransduction ion channel blockers. FM1-43, streptomycin, and 2-APB all reduce the transduction current compared with that recorded in the absence of blockers. For ***C***, median and interquartile ranges are overlaid on the data points. ***D***, Dose–response curve of pymetrozine action on the transduction current amplitude.

The dendrites of Group III neurons ranged from 80 to 176 μm in length (Fig. 7*A*,*B*; neurons labeled * and +), but there was no correlation between the length and the magnitude of the transduction current as measured under voltage clamp at the soma, when the spikes were blocked (Fig. 7*B*). We speculate that the number of transduction ion channels and the resulting tone-evoked current is larger for neurons with longer dendrites. The adapted transduction current median amplitude (measured at the end of the 250 ms sound stimulus; Fig. 6*E*) under standard saline was 144 pA (*n* = 20, *N* = 18).

The transduction current is attenuated due to the cable properties of the dendrite. The length constant was calculated using the membrane resistance and capacitance of the space clamped region, with an assumed membrane capacitance of 1 μF/cm^2^ and axoplasmic resistance of 132 Ω/m (Yarom, 1978). The dendrite diameter was measured directly from confocal images at the midpoint between the soma and the dendrite dilation. Based on the estimated length constant (220 ± 83 μm; *n* = 20, *N* = 20) and the measured dendrite length (117 ± 23 μm; *n* = 20, *N* = 20), the transduction current at the cilium was, on average, 347 ± 196 pA (approximately three times higher than the transduction current at the soma). This value was used to calculate the single channel current of the transduction ion channels based on the following: (1) the conductance of candidate mechanotransduction ion channels expressed in heterologous cells; and (2) the predicted transmembrane potential across the ciliary membrane of 130 mV (a combination of the positive receptor lymph cavity, assumed to be +60 mV, and the −70 mV resting potential of the auditory neurons measured here). The single channel conductance of *Drosophila* NompC when expressed heterologously is 140 pS (Yan et al., 2013), which leads to a tentative estimate of 18 pA for its single channel current in the cilium. The single channel conductance for Nanchung-Inactive is unknown, but other mammalian TRPV channels have conductances of up to 90 pS (Strotmann et al., 2000), which gives an estimated single channel current of 12 pA in the cilium.

A range of ion channel blockers, which are known to also block mechanotransduction ion channels, were used to pharmacologically characterize the transduction ion channel (for details, see Materials and Methods). About 5 min after whole-cell recordings were established, there was detectable rundown of the transduction current such that it was abolished after ∼10 min. The transduction current was therefore measured directly after breakthrough to whole-cell conditions. Four of seven mechanotransduction ion channel blockers failed to block the transduction current (Fig. 7*C*): gadolinium (*n* = 13, *N* = 10; median, 150 pA; *p* > 0.999); amiloride (*n* = 11, *N* = 7; median, 138 pA); Ruthenium red (*n* = 9, *N* = 3; median, 177 pA; *p* > 0.999); SKF-96365 (*n* = 13, *N* = 4; median, 126 pA; *p* = 0.337; one-way ANOVA with Sidak's multiple-comparisons test). The blockers FM1-43, streptomycin, and 2-APB had a strong blocking effect on the transduction current [Fig. 7*C*; *n* = 14, *N* = 11; *n* = 11, *N* = 8; *n* = 10, *N* = 7; median, 67, 29, and 26 pA, respectively; one-way ANOVA, *p* = 0.007, *p* < 0.0001, *p* < 0.0001, respectively; Bonferroni's multiple-comparisons test, with degrees of freedom 1 = 7 (between group) and degrees of freedom 2 = 93 (within group), was used for all ANOVAs for mechanotransduction channel blockers]. Pymetrozine specifically targets chordotonal organ neurons (Ausborn et al., 2005) through its agonistic action on Nanchung-Inactive ion channels (Nesterov et al., 2015). The EC_50_ for pymetrozine acting on Nanchung-Inactive expressed in CHO cells was 100 nm (Nesterov et al., 2015). We quantified the ability of pymetrozine to open Nanchung-Inactive channels in locust auditory neurons by generating a dose–response curve (Fig. 7*D*) and determining the EC_50_, which was 21 nm.

### Role of Nanchung-Inactive in generating the transduction current

**Figure 8.**
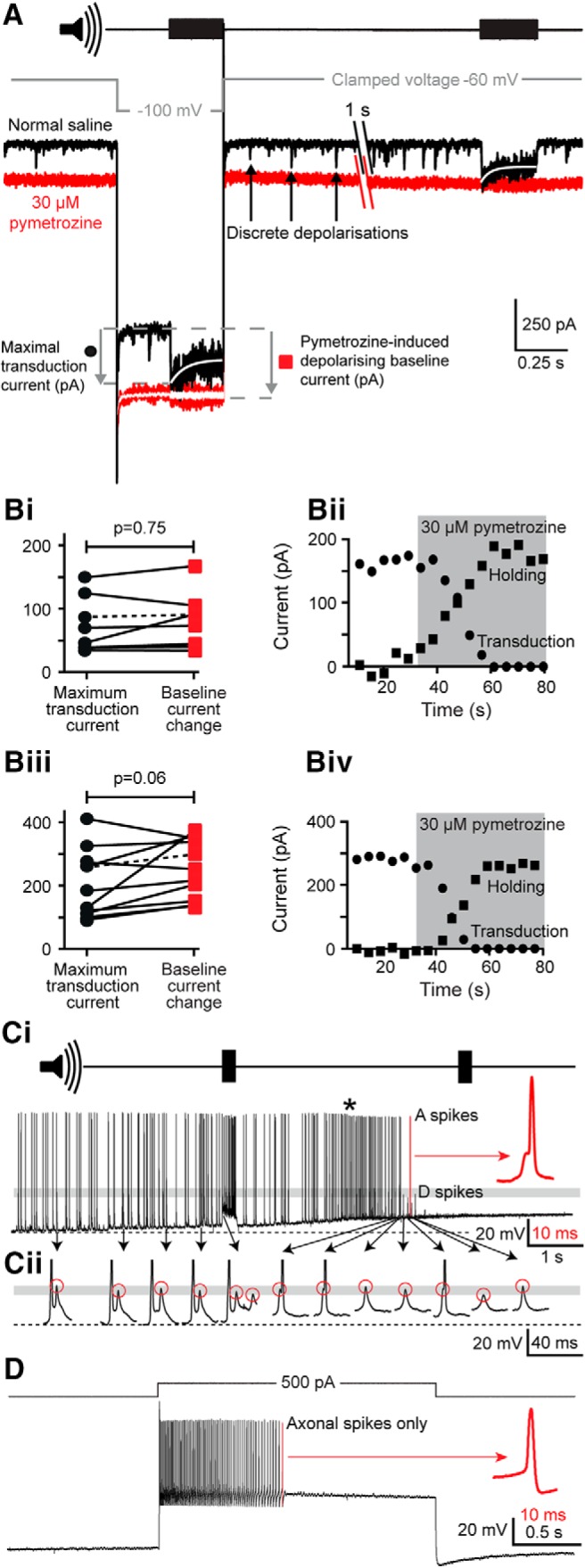
The effect of pymetrozine on baseline current, maximal transduction current, and spiking activity. ***A***, Characterization of the baseline current and maximal transduction currents before (black) and after perfusion of 30 μm pymetrozine (red) using the voltage step and sound stimulus protocol introduced in Figure 6*E*. In the presence of 90 nm TTX and 20 mm TEA, the application of pymetrozine induced a larger baseline current at rest (−60 mV) and at a clamped voltage of −100 mV. Pymetrozine also abolished the tone-induced transduction current measured at −100 mV and at −60 mV. The transduction currents were fitted with a single order exponential (white curves). The amplitude of the fit 5 ms after tone onset was used as a measure of the maximal transduction current. The white trace overlaying the (red) current trace, after pymetrozine application at −100 mV, is an average of eight presentations of the tone revealing no residual tone-evoked currents. ***Bi***, Relationship between the maximal transduction current measured under control conditions (black circles) and the baseline current shift at −60 mV measured 30 s after the application of 30 μm pymetrozine (red squares) in the presence of TTX and TEA. Solid lines link pairs of measurements made from the same neuron. The dashed line indicates the example shown in ***Bii*** (*n* = 10, *N* = 10). ***Bii***, Time course of pymetrozine action on the transduction current (circles) and the holding current (squares) at −60 mV; pymetrozine application is indicated by the gray-shaded region. ***Biii***, As in ***Bi***, but the maximal transduction current and baseline current shift were measured at −100 mV. ***Biv***, As in ***Bii***, but the time course of pymetrozine action on the transduction current and holding current were measured at −100 mV. ***Ci***, Application of pymetrozine transiently increases spontaneous spike frequency (asterisk, measured in current clamp) before abolishing both spontaneous spikes and sound-evoked transduction potentials (no response to second sound pulse, top trace). All axonal (A) spikes arise from dendritic (D) spikes (e.g., inflection marked by red arrow, red expanded spike). Dashed line, resting membrane potential. ***Cii***, The peak depolarization of the dendritic spikes (red circles) are unaffected by pymetrozine (all lie within the gray band). The axonal spikes are truncated for clarity. ***D***, After pymetrozine application, axonal spikes (but not dendritic spikes) can still be elicited by current injection. They lack any inflection (red arrow).

A specific agonist, pymetrozine, was used to examine the potential contribution of the heteromeric mechanotransduction ion channel candidate Nanchung-Inactive to the auditory transduction current in Müller's organ. We used the same transduction current isolation protocol as illustrated in Figure 6*E* to measure the maximal transduction current. Perfusion of 30 μm pymetrozine (Fig. 8*A*, red trace) elicited a sustained depolarizing current of 162 ± 74 pA at −60 mV holding potential, and 256 ± 92 pA at −100 mV holding potential relative to the control saline (Fig. 8*A*, black trace). It also caused the discrete depolarizing potentials to disappear (Fig. 8*A*, black arrows). Crucially, it also abolished the transduction current elicited by a tone presented while neurons were clamped at −100 mV (Fig. 8*A*) or at −60 mV. To compare the amplitude of the maximal tone-evoked transduction current (before pymetrozine application) with that of the pymetrozine-induced change in baseline current, we fitted the adapting transduction current with a single-order exponential (Fig. 8*A*, white curves) to remove noise and determined the maximal sound-evoked transduction current 5 ms after tone onset, when it was maximal. The increase in depolarizing baseline current recorded during pymetrozine application (difference between black and red traces) was comparable in amplitude to the maximal tone-evoked transduction current recorded in the absence of pymetrozine (Fig. 8*A*, black trace during tones, gray dashed lines and black circle; also see next paragraph for quantification).

We measured both the maximal tone-evoked transduction current and the pymetrozine-induced shift in depolarizing baseline current at both −60 and −100 mV. The median maximal tone-evoked transduction current before perfusion of pymetrozine at −60 and −100 mV was 114 and 183 pA (mean ± SD: 146 ± 88 and 206 ± 106 pA). The introduction of pymetrozine into the saline resulted in a depolarization of the baseline current at −60 and −100 mV (Fig. 8A,*Bi*,*Biii*, red squares) of 163 and 252 pA (mean ± SD: 160 ± 89 and 255 ± 92 pA). Student's paired two-tailed *t* tests provided no evidence that this difference (between the amplitude of the tone-evoked transduction current before pymetrozine application and the pymetrozine-induced tonic depolarization) was real at −60 mV (*p* = 0.75; Fig. 8*Bi*) and only weak evidence that the difference was real at −100 mV (*p* = 0.0613; Fig. 8*Biii*). After pymetrozine application and abolition of the transduction current, we detected no residual tone-evoked depolarization even when we increased our signal-to-noise ratio by averaging eight traces for each of the 11 recordings (Fig. 8*A*, white trace overlaid on red pymetrozine trace at −100 mV). Our limit of detectability was 10 pA [based on a signal-to-noise (root mean squared = 4.78 pA) ratio of 2:1]. Thus, we would not be able to detect a residual transduction current of 10 pA at our somatic recording site, which equates to ∼30 pA at the cilium based on our estimate of the dendritic length constant.

Following the application of pymetrozine, the maximal transduction current (Fig. 8*Bii*,*Biv*, circles) decreased with a time course that matched the increase in depolarizing baseline current (Fig. 8*Bii*,*Biv*, squares) at both −60 and −100 mV. After ∼30 s of pymetrozine application, the transduction current was abolished and the depolarizing baseline current reached its full magnitude (Fig. 8*A*,*Bii*,*Biv*). The opening of Nanchung-Inactive channels can therefore explain the entire tone-evoked transduction current.

We examined the physiological effect of pymetrozine-evoked depolarization on spike generation and the transduction potential in current-clamp recordings (Fig. 8*C*). Pymetrozine application first led to depolarization of the neuron within a few seconds, with an initial increase in spontaneous spiking (Fig. 8*Ci*, asterisk). This was rapidly followed by the abolition of both axonal and dendritic spontaneous spikes, tone-evoked spikes, and the tone-evoked transduction potential (Fig. 8*Ci*). Spikes before and during pymetrozine application rode on a dendritic spike inflection (Fig. 8*Ci*, red arrow). The peak depolarization values of dendritic spikes before and after pymetrozine-induced depolarization were similar (Fig. 8*Cii*). After pymetrozine application, axonal spikes could be triggered only by current injection, and they lacked the inflection due to dendritic spikes (Fig. 8*D*, red arrow).

**Figure 9.**
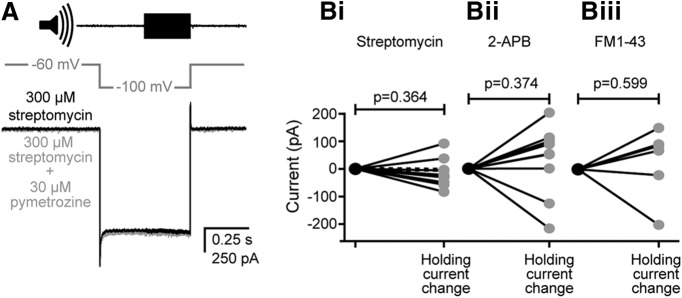
The effect of pymetrozine on the baseline current when the transduction current is blocked with preapplication of streptomycin, 2-APB, or FM1-43. ***A***, The baseline current before (black) and 60 s after perfusion of 30 μm pymetrozine (gray), with 15 min preapplication of 300 μm streptomycin (which completely blocks the tone-evoked transduction current). ***Bi***, Quantification of the effect of 30 μm pymetrozine on the baseline current (black) 60 s after pymetrozine perfusion with preapplication of 300 μm streptomycin (dashed line indicates the example in ***A***; *n* = 10, *N* = 10). ***Bii***, Quantification of the effect of 30 μm pymetrozine on the baseline current (black) 60 s after pymetrozine perfusion with preapplication of 300 μm 2-APB (*n* = 10, *N* = 10). ***Biii***, Quantification of the effect of 30 μm pymetrozine on the baseline current (black) 60 s after pymetrozine perfusion with preapplication of 9 μm FM1-43 [*n* = 6, *N* = 6; a residual tone-evoked transduction current was present in 4 of 10 recordings (indicating incomplete block by FM1-43), which were therefore excluded from this analysis].

To examine whether antagonist-mediated block of auditory transduction (Fig. 7*C*) could be explained by the block of Nanchung-Inactive channels, 30 μm pymetrozine was applied after the tone-evoked transduction current was already blocked by 300 μm streptomycin (Fig. 9*A*), 300 μm 2-APB, or 9 μm FM1-43. Pymetrozine caused no change in the baseline depolarizing current (measured at −100 mV, where baseline shift was maximal) for any of the three antagonists (Fig. 9*Bi–Biii*). The mean changes in holding current for preparations blocked with streptomycin, 2-APB, or FM1-43 were as follows: −16 ± 50, −36 ± 122, or 28 ± 125 pA, respectively; Student's paired two-tailed *t* test, *p* = 0.364, *p* = 0.374, *p* = 0.600, respectively).

## Discussion

Chordotonal organ neurons are monodendritic, ciliated, mechanosensitive cells that can function as proprioceptors and in the reception of external stimuli, such as vibration, gravity, and sound (Field and Matheson, 1998; Kamikouchi et al., 2009). This study has pioneered whole-cell patch-clamp recordings from auditory chordotonal neurons of Müller's organ of the desert locust to understand the process of mechanotransduction in hearing. We show that both dendritic and axonal spikes rely on voltage-gated sodium channels, and use ion channel blockers and a Nanchung-Inactive-specific agonist to investigate auditory transduction.

### Axonal and dendritic spikes

Mechanotransduction in chordotonal organ auditory neurons in Orthoptera involves the following: (1) the conversion of mechanical displacements into a transduction potential at their monodendritic ciliated end; (2) the initiation of dendritic spikes by the transduction potential; and (3) the triggering of axonal spikes by dendritic spikes. In the initial step, nanometer displacements of the tympanum, onto which Müller's organ neurons are attached, are transduced by the opening of mechanically gated ion channels in the membrane of the cilium. The postulated high electrochemical gradient across the ciliary membrane drives cations such as potassium and calcium into the cilium (Küppers, 1974; Thurm and Küppers, 1980), and the resulting depolarization triggers dendritic spikes—the second step.

Concurrent intracellular recordings from the auditory neurons and their distal attachment cells, into which their cilia insert, convincingly demonstrated that the dendritic spikes are initiated within the distal segment of the neuron (Oldfield and Hill, 1986). Could the dendritic spikes be ciliary spikes, carried by Nanchung-Inactive along the proximal cilium as predicted by the NompC model (Göpfert et al., 2006)? It is assumed that the scolopale lymph protects the distal dendrite and cilium from external changes in ion concentrations (Roy et al., 2013), so our demonstration that extracellular sodium can reversibly reduce and abolish dendritic spikes implies that they are not restricted to the distal dendrite or cilium but instead propagate along the length of the dendrite. The block of dendritic spikes with TTX and their voltage-gated nature suggests that they are carried by voltage-gated sodium channels. Finally, to test whether Nanchung-Inactive contributed to dendritic spikes, we opened the channels with pymetrozine. If dendritic spikes were carried by Nanchung-Inactive ion channels in the cilium, they should be abolished or reduced. However, after pymetrozine-induced depolarization, we still detected dendritic spikes of similar amplitude to those recorded in the absence of pymetrozine (Fig. 8*Cii*).

In the third step, dendritic spikes trigger axonal spikes. The larger (basal) spikes were thought to initiate in the dendrite base (Hill, 1983b; Oldfield and Hill, 1986), but our finding that dendritic spikes propagate along the dendrite suggests that the larger (basal) spikes might initiate previously. We suggest that larger (basal) spikes are triggered in the initial axon segment and have called them axonal spikes. Upon prolonged acoustic stimulation, we noticed repolarization after an dendritic spike but before the threshold of the axonal spike was triggered (Fig. 3*Bii*), which we interpreted as the dendritic spike traveling past our recording site at the soma before triggering an axonal spike in the initial axon segment.

We suggest that the difference in amplitude between the small dendritic and large axonal spikes (21 and 75 mV measured in this study; 25 and 65 mV measured in the study by Hill, 1983b) is not due to a difference in the extracellular ionic concentrations between the receptor lymph enclosed by the scolopale cell and that outside the receptor lymph cavity (Hill, 1983b; Oldfield and Hill, 1986), but is due a standing potassium current that enters through the cilium. This would lead to an increased potassium-driven repolarization within the dendrite that would counter sodium-driven depolarization to decrease spike amplitude.

Dendritic spikes triggered by the receptor potential mirror the mechanism described for a spider vibration-sensitive slit sense organ neuron, where depolarization at the ciliated end is actively propagated along the ∼150 μm dendrite by voltage-gated sodium channels (Seyfarth et al., 1995). The dendrites of chordotonal organ neurons of Diptera are considerably shorter (∼15 μm; Karak et al., 2015; Andrés et al., 2016) than the dendrites of orthopteran chordotonal neurons, which are ∼50 μm for cricket (Oldfield and Hill, 1986) and ∼110 μm for locust auditory neurons (measured here). Thus, dendritic spikes might not be a general principle of operation of chordotonal organ neurons, but rather a specialization for long dendrites.

### Transduction current and the number of transduction channels

In the absence of acoustic stimulation, insect auditory neurons exhibit sporadic discrete depolarizations of varying amplitude, which we interpreted as stochastic opening of mechanotransduction ion channels (Hill, 1983a; Oldfield and Hill, 1986). Upon acoustic stimulation, these discrete depolarizations summate to produce a graded transduction potential. In auditory neurons of vertebrates and flies, the mechanotransduction ion channels are thought to flicker between open and closed states to maximize their sensitivity to incoming sound by maintaining an open probability close to 0.5 (Nadrowski et al., 2004, 2008); this results in noisy transduction currents in vertebrate hair cells (Holton and Hudspeth, 1986). At 21°C, the channel open probability measured here is well <0.5, but the strong temperature dependence of the channels means that this may be an underestimate compared with the higher temperatures (36°C during daytime) in which they are reared. Our estimate of the number of transduction ion channels suggests that there are between 19 and 29 mechanotransduction channels per auditory neuron; about a quarter that of vertebrate hair cells (Ricci et al., 2003) and three to four times higher than that predicted for individual auditory neurons of Johnston's organ in the fly (Albert et al., 2007).

### The role of Nanchung-Inactive in producing the mechanotransduction current

The two TRP ion channels NompC and Nanchung-Inactive form ion channels when expressed in heterologous cells (Gong et al., 2004; Yan et al., 2013); are localized to the cilium (Kim et al., 2003; Gong et al., 2004; Lee et al., 2010), where transduction is postulated to take place; and reduce the transduction current when knocked out (Lehnert et al., 2013). Both are therefore candidate mechanotransduction ion channels in insect auditory neurons (Göpfert et al., 2006; Lehnert et al., 2013), as proposed in the NompC model or the Nanchung-Inactive model (Albert and Göpfert, 2015). The role of either in producing the mechanotransduction current is not known. Our pharmacological approach revealed that the general ion channel blockers (also known to block mechanotransduction ion channels) SKF-96365, gadolinium, and Ruthenium red all failed to block the transduction current in locust auditory neurons. The mechanotransduction current in locust auditory neurons is blocked by FM1-43, which is known to block NompC in heterologous cells (Yan et al., 2013). The transduction current was also blocked by 2-APB, a general TRP channel blocker and modulator, and streptomycin, which is predicted to block the permeation pore of the (unidentified) transduction ion channel in vertebrate auditory neurons (Marcotti et al., 2005; van Netten and Kros, 2007). By themselves, these blockers are not specific enough to identify the channel. However, their block combined with the effects of pymetrozine, allows us to determine whether they block Nanchung-Inactive ion channels. Pymetrozine is thought to be a highly specific agonist for Nanchung-Inactive channels in a 1:1 stoichiometry (Nesterov et al., 2015) because it does not affect the responses of the two other insect mechanoreceptor types: bristles and campaniform (Ausborn et al., 2005), which contain other candidate transduction ion channels such as NompC (Lee et al., 2010; Liang et al., 2011).

First, we demonstrated that pymetrozine alone induced a depolarizing current that was comparable in amplitude to the maximal sound-evoked transduction current. Thus, the Nanchung-Inactive channels, known to be located in the proximal cilium (Gong et al., 2004), are able to pass all the current necessary to explain the maximal auditory transduction current. We further demonstrated that in the presence of pymetrozine there was no additional tone-evoked depolarization, which allows us to calculate an upper limit of 30 pA for any possible remaining transduction currents. We went on to use pymetrozine to examine whether the action of streptomycin, 2-APB, and FM1-43 was due to the block of Nanchung-Inactive. After block of the auditory transduction current with streptomycin, 2-APB, or FM1-43, the application of pymetrozine now failed to elicit any depolarization, providing strong evidence that all three block Nanchung-Inactive. This is not proof that Nanchung-Inactive is, or forms part of, the mechanotransduction ion channel because FM1-43 blocks NompC in heterologous cells, and it is possible that all three blockers (FM1-43, 2-APB, and streptomycin) block NompC, or another unidentified channel, in locust auditory neurons. Thus, this purely pharmacological approach does not rule out the involvement of another mechanotransduction ion channel upstream that acts to open Nanchung-Inactive channels in the proximal cilium. If NompC is the upstream channel, we estimate only one to two NompC channels at the tips of locust auditory neurons, which is below the six channels predicted for *Drosophila* auditory neurons (Albert et al., 2007).

Nanchung-Inactive could be, or form part of, the primary mechanotransduction ion channel of locust auditory neurons, which is in agreement with recordings of *Drosophila* transduction currents (Lehnert et al., 2013); if not, our recordings establish an upper limit of upstream transduction currents that trigger Nanchung-Inactive channel opening.

## References

[B1] Albert JT, Göpfert MC (2015) Hearing in *Drosophila*. Curr Opin Neurobiol 34:79–85. 10.1016/j.conb.2015.02.001 25710304 PMC4582067

[B2] Albert JT, Nadrowski B, Göpfert MC (2007) Mechanical signatures of transducer gating in the *Drosophila* ear. Curr Biol 17:1000–1006. 10.1016/j.cub.2007.05.004 17524645

[B3] Andrés M, Seifert M, Splathoff C, Warren B, Weiss L, Giraldo D, Winkler M, Pauls S, Göpfert MC (2016) Auditory efferent system modulates mosquito hearing. Curr Biol 26:2028–2036. 10.1016/j.cub.2016.05.077 27476597

[B4] Ausborn J, Wolf H, Mader W, Kayser H (2005) The insecticide pymetrozine selectively affects chordotonal mechanoreceptors. J Exp Biol 208:4451–4466. 10.1242/jeb.01917 16339866

[B5] Cheng LE, Song W, Looger LL, Jan LY, Jan YN (2010) The role of the TRP channel NompC in *Drosophila* larval and adult locomotion. Neuron 67:373–380. 10.1016/j.neuron.2010.07.004 20696376 PMC2933178

[B6] Effertz T, Wiek R, Göpfert MC (2011) NompC TRP channel is essential for *Drosophila* sound receptor function. Curr Biol 21:592–597. 10.1016/j.cub.2011.02.048 21458266

[B7] Field LH, Matheson T (1998) Chordotonal organs of insects. In: Advances in insect physiology, Vol 27 (Evans PD, ed), pp 2–230. San Diego, London: Academic.

[B8] Gong J, Wang Q, Wang Z (2013) NOMPC is likely a key component of *Drosophila* mechanotransduction channels. Eur J Neurosci 38:2057–2064. 10.1111/ejn.12214 23590241

[B9] Gong Z, Son W, Chung YD, Kim J, Shin DW, McClung CA, Lee Y, Lee HW, Chang DJ, Kaang BK, Cho H, Oh U, Hirsh J, Kernan MJ, Kim C (2004) Two interdependent TRPV channel subunits, inactive and nanchung, mediate hearing in *Drosophila*. J Neurosci 24:9059–9066. 10.1523/JNEUROSCI.1645-04.2004 15483124 PMC6730075

[B10] Göpfert MC, Albert JT, Nadrowski B, Kamikouchi A (2006) Specification of auditory sensitivity by *Drosophila* TRP channels. Nat Neurosci 9:999–1000. 10.1038/nn1735 16819519

[B11] Gray EG (1960) The fine structure of the insect ear. Philos Trans R Soc Lond B Biol Sci 243:75–94. 10.1098/rstb.1960.0005

[B12] Hill KG (1983a) The physiology of locust auditory receptors I. discrete depolarisations of receptor cells. J Comp Physiol 152:475–482. 10.1007/BF00606437

[B13] Hill KG (1983b) The physiology of locust auditory receptors II. membrane potentials associated with the response of the receptor cell. J Comp Physiol 152:483–493. 10.1007/BF00606438

[B14] Holton T, Hudspeth AJ (1986) The transduction channel of hair cells from the bull-frog characterized by noise analysis. J Physiol 375:195–227. 10.1113/jphysiol.1986.sp016113 2432221 PMC1182755

[B15] Jacobs K, Otte B, Lakes-Harlan R (1999) Tympanal receptor cells of *Schistocerca gregaria*: correlation of soma positions and dendrite attachment sites, central projections and physiologies. Dev Neurobiol 283:270–285.

[B16] Kamikouchi A, Inagaki HK, Effertz T, Hendrich O, Fiala A, Göpfert MC, Ito K (2009) The neural basis of *Drosophila* gravity-sensing and hearing. Nature 458:165–171. 10.1038/nature07810 19279630

[B17] Kang L, Gao J, Schafer WR, Xie Z, Xu XZ (2010) C. elegans TRP family protein TRP-4 is a pore forming subunit of a native mechanotransduction channel. Neuron 67:381–391. 10.1016/j.neuron.2010.06.032 20696377 PMC2928144

[B18] Karak S, Jacobs JS, Kittelmann M, Spalthoff C, Katana R, Sivan-Loukianova E, Schon MA, Kernan MJ, Eberl DF, Göpfert MC (2015) Diverse roles of axonemal dyneins in *Drosophila* auditory neuron function and mechanical amplification in hearing. Sci Rep 5:17085. 10.1038/srep17085 26608786 PMC4660584

[B19] Kemp DT (2002) Otoacoustic emissions, their origin in cochlear function, and use. Br Med Bull 63:223–241. 10.1093/bmb/63.1.223 12324396

[B20] Kim J, Chung YD, Park DY, Choi S, Shin DW, Soh H, Lee HW, Son W, Yim J, Park CS, Kernan MJ, Kim C (2003) A TRPV family ion channel required for hearing in *Drosophila*. Nature 424:81–84. 10.1038/nature01733 12819662

[B21] Kössl M, Boyan GS (1998) Otoacoustic emissions from a nonvertebrate ear. Naturwissenschaften 85:124–127. 10.1007/s001140050467 9562992

[B22] Küppers J (1974) Measurements on the ionic milieu of the receptor terminal in mechanoreceptive sensilla of insects. In: Symposium mechanoreception (Schwartzkopff J, ed), pp 387–394. Wiesbaden, Germany: VS Verlag für Sozialwissenschaften.

[B23] Lee J, Moon S, Cha Y, Chung YD (2010) *Drosophila* TRPN (=NOMPC) channel localizes to the distal end of mechanosensory cilia. PLoS One 5:e11012. 10.1371/journal.pone.0011012 20543979 PMC2882365

[B24] Lehnert BP, Baker AE, Gaudry Q, Chiang AS, Wilson RI (2013) Distinct roles of TRP channels in auditory transduction and amplification in *Drosophila*. Neuron 77:115–128. 10.1016/j.neuron.2012.11.030 23312520 PMC3811118

[B25] Liang X, Madrid J, Saleh HS, Howard J (2011) NompC, a member of the TRP channel family, localizes to the tubular body and distal cilium of *Drosophila* campaniform and chordotonal receptor cells. Cytoskeleton 68:1–7. 10.1002/cm.20493 21069788 PMC3048163

[B26] Marcotti W, van Netten SM, Kros CJ (2005) The aminoglycoside antibiotic dihydrostreptomycin rapidly enters mouse outer hair cells through the mechano-electrical transducer channels. J Physiol 567:505–521. 10.1113/jphysiol.2005.085951 15994187 PMC1474200

[B27] Möckel D, Seyfarth EA, Kössl M (2007) The generation of DPOAEs in the locust ear is contingent upon the sensory neurons. J Comp Physiol A Neuroethol Sens Neural Behav Physiol 193:871–879. 10.1007/s00359-007-0239-5 17534628

[B28] Nadrowski B, Martin P, Jülicher F (2004) Active hair-bundle motility harnesses noise to operate near an optimum of mechanosensitivity. Proc Natl Acad Sci U S A 101:12195–12200. 10.1073/pnas.0403020101 15302928 PMC514456

[B29] Nadrowski B, Albert JT, Göpfert MC (2008) Transducer-based force generation explains active process in *Drosophila* hearing. Curr Biol 18:1365–1372. 10.1016/j.cub.2008.07.095 18789690

[B30] Narahashi T, Moore JW, Scott WR (1964) Tetrodotoxin blockage of sodium conductance increase in lobster giant axons. J Gen Physiol 47:965–974. 10.1085/jgp.47.5.965 14155438 PMC2195365

[B31] Nesterov A, Spalthoff C, Kandasamy R, Katana R, Rankl NB, Andrés M, Jähde P, Dorsch JA, Stam LF, Braun FJ, Warren B, Salgado VL, Göpfert MC (2015) TRP channels in insect stretch receptors as insecticide targets. Neuron 86:665–671. 10.1016/j.neuron.2015.04.001 25950634

[B32] Nowotny M, Hummel J, Weber M, Möckel D, Kössl M (2010) Acoustic-induced motion of the bushcricket (*Mecopoda elongata*, Tettigoniidae) tympanum. J Comp Physiol A Neuroethol Sens Neural Behav Physiol 196:939–945. 10.1007/s00359-010-0577-6 20827480

[B33] Oldfield BP, Hill KG (1986) Functional organization of insect auditory sensilla. J Comp Physiol A Neuroethol Sens Neural Behav Physiol 158:27–34. 10.1007/BF00614517

[B34] Ricci AJ, Crawford AC, Fettiplace R (2003) Tonotopic variation in the conductance of the hair cell mechanotransducer channel. Neuron 40:983–990. 10.1016/S0896-6273(03)00721-9 14659096

[B35] Roy M, Sivan-Loukianova E, Eberl DF (2013) Cell-type-specific role of Na^+^/K^+^ ATPase subunits in *Drosophila* auditory mechanosensation. Proc Natl Acad Sci U S A 110:181–186. 10.1073/pnas.1208866110 23248276 PMC3538205

[B36] Seyfarth EA, Sanders EJ, French AS (1995) Sodium channel distribution in a spider mechanosensory organ. Brain Res 683:93–101. 10.1016/0006-8993(95)00317-J 7552349

[B37] Strotmann R, Harteneck C, Nunnenmacher K, Schultz G, Plant TD (2000) OTRPC4, a nonselective cation channel that confers sensitivity to extracellular osmolarity. Nat Cell Biol 2:695–702. 10.1038/35036318 11025659

[B38] Thurm U, Küppers J (1980) Epithelial physiology of insect sensilla. In: Insect biology of the future (Löcke M, Smith DS, eds), pp 735–763. New York, London: Academic.

[B39] van Netten SM, Kros CJ (2007) Insights into the pore of the hair cell transducer channel from experiments with permeant blockers. Curr Top Membr 59:375–398. 10.1016/S1063-5823(06)59013-1 25168143

[B40] Yan Z, Zhang W, He Y, Gorczyca D, Xiang Y, Cheng LE, Meltzer S, Jan LY, Jan YN (2013) Drosophila NOMPC is a mechanotransduction channel subunit for gentle-touch sensation. Nature 493:221–225. 10.1038/nature11685 23222543 PMC3917554

[B41] Yarom Y (1978) Physiological and morphological differentiation of giant axons during the development of the cockroach Periplaneta americana. PhD thesis, Hebrew University.

